# Coming Soon to a Class Near You: Cinematic Insights on Later Life

**DOI:** 10.1093/geroni/igab046.266

**Published:** 2021-12-17

**Authors:** Candace Brown, Margaret Perkinson

**Affiliations:** 1 University of North Carolina, Charlotte, Charlotte, North Carolina, United States; 2 University of Hawaii at Manoa, Honolulu, Hawaii, United States

## Abstract

Cinema can enhance gerontological education by reinforcing a variety of learning styles, connecting course content to current culture, and providing an alternative, tangible view of what students are learning. The presenters discuss their use of film to teach gerontological concepts in the classroom. In an Introduction to Aging course, the films, “Young at Heart” and “Sunset Story” were used to break through ageist stereotypes, examine examples of resilience at the end of life, and convey the impact of residential context on the experience of aging, i.e., within a retirement home for “retired rebels.” In a course on Health and Aging, movies, such as “Red,” “Driving Miss Daisy,” and “Somethings Gotta Give” are used to compare the social and psychological aspects of aging of the characters to learned concepts in the classroom. Students expressed how watching and writing about the films increased their understanding by bringing abstract gerontological concepts to life.

